# Voices of change: associations between vocal markers and symptoms of ADHD - Findings from the LIFE child study

**DOI:** 10.1007/s00787-025-02954-9

**Published:** 2026-02-26

**Authors:** Rachel Bamberger, Leon D. Lotter, Nicolás Nieto, Tanja Poulain, Antje Körner, Wieland Kiess, Michael Fuchs, Georg von Polier

**Affiliations:** 1 Institute of Neuroscience and Medicine, Brain & Behaviour (INM-7), Research Centre Jülich, Jülich, Germany; 2 Department of Child and Adolescent Psychiatry, Psychotherapy and Psychosomatics, University Hospital RWTH Aachen, Aachen, Germany; 3 Max Planck School of Cognition, Stephanstrasse 1A, Leipzig, 04103 Germany; 4 Institute of Systems Neuroscience, Medical Faculty, Heinrich Heine University, Düsseldorf, Germany; 5 Department of Pediatrics, Center for Pediatric Research Leipzig (CPL), Medical Faculty, Leipzig University, Leipzig, Germany; 6 LIFE Child Leipzig Research Center for Civilization Diseases, Medical Faculty, Leipzig University, Leipzig, Germany; 7 German Center for Child and Adolescent Health (DZKJ), partner site Leipzig/Dresden, Leipzig, Germany; 8 Helmholtz Institute of Metabolic, Obesity & Vascular Research (HI-MAG), Helmholtz Munich and the University of Leipzig, Leipzig, Germany; 9 Section of Phoniatrics and Audiology, Department of Otorhinolaryngology, University Hospital Leipzig, Leipzig, Germany; 10 Department of Child and Adolescent Psychiatry, Psychotherapy and Psychosomatics, University Hospital Leipzig, Leipzig, Germany

**Keywords:** Precision psychiatry, ADHD, Voice, Machine learning, Prosody

## Abstract

In the context of precision psychiatry, voice characteristics in psychiatric disorders have significant potential as diagnostic markers. However, most studies have focused on adults, resulting in a poor understanding of paediatric voice characteristics. The diagnosis of *attention-deficit/hyperactivity disorder (ADHD)* lacks objectivity and could potentially benefit from the inclusion of voice features as objective markers. This study used linear mixed models and machine learning to investigate associations between voice characteristics and parent-reported symptoms of hyperactivity and inattention in a population-based cohort. Two tasks were used to derive speaking-voice characteristics: counting and sustaining a tone. 2418 recordings from 1460 children of the LIFE Child cohort aged 5–18 years (49% girls) were included. We analysed demographic and health data, voice parameters, and hyperactivity/inattention scores from the *Strengths and Difficulties Questionnaire* (SDQ). Results indicated linear associations between voice features, particularly fundamental frequency and voice intensity, and SDQ scores. Machine learning models predicted hyperactivity/inattention scores with moderate accuracy (r = 0.36). Predictive performance was higher in girls than in boys, an effect largely attributable to overall lower symptom ratings by parents in girls. Our findings support the inclusion of voice characteristics in research on diagnostic markers in paediatric populations and underline the need for further studies to refine analytic approaches.

**Supplementary Information:**

The online version contains supplementary material available at 10.1007/s00787-025-02954-9.

## Introduction

At a time when around one in four children experience mental health problems [1, 2], *precision psychiatry* is gaining attention as promising approach to personalised care. The use of preventive, diagnostic, and therapeutic methods adapted to individual patients’ characteristics has been studied for more than two decades [3]. Precision psychiatry aims to individualise mental health care e.g. by increasing accuracy of diagnosis and treatment [4]. In the context of child and adolescent psychiatry, efforts have focused on developing more accurate and precise diagnostic or risk stratification tools to tackle current, largely subjective, diagnostic practice [5]. One approach to identifying objective markers is breaking down disorders into their underlying pathophysiological features [6], which can be detected through measuring biosignals [7].

Acoustic biosignals are gaining recognition as tools to study pathophysiological mechanisms of psychiatric disorders [8–10]. Voice features may contain essential information about an individual’s state and potentially indicate pathologies [7, 11]. Human voice is produced by complex interactions between the cognitive and neuromuscular system, making voice a highly sensitive output system [7]. Current research in adults indicates decreases in the fundamental frequency (f0, the basic pitch of the voice) and f0 range in individuals with major depressive disorder, which can result in monotonous intonation and a deeper voice [12]. In anxiety disorders, studies have found a significant increase in the f0 [12]. A recent scoping review points to the potential of paediatric voice as a promising method for the detection of several childhood conditions and disorders, such as autism spectrum disorder [13]. If voice features were translated into health-relevant indices, they could eventually become part of a more objective assessment within *biomarker batteries* [11].

One promising field of voice related biomarker research is attention-deficit/hyperactivity disorder (ADHD): Machine learning (ML) based studies in adults with ADHD have demonstrated the predictive utility of voice features for ADHD classification, as well as associations with symptom severity [14]. Biologically, ADHD is linked to changes in the brain’s dopamine signals, which impact motor control [15]. Voice production as well is linked to dopamine levels and involves complex motor behaviour. Thus, changes in dopamine pathways related to ADHD may alter vocal patterns in people with ADHD [16]. Children with ADHD seem to show altered voice features (e.g., f0, intensity, jitter) and more hyperfunctional vocal behaviour (e.g., more shouting; excessive, louder, faster talking) in comparison to their peers [17, 18]. These bodily changes make children with ADHD prone to voice disorders [19, 20]. Investigating this component of ADHD pathophysiology, in the form of voice features, could provide new insights and enrich the current state of knowledge in child and adolescent precision psychiatry [5, 21].

Our population-based study investigated children’s voice features that may serve as indices of ADHD using multivariate statistics, including linear mixed models (LMM) and machine learning (ML) approaches. We aimed to evaluate the association of different acoustic parameters obtained under several vocal tasks with symptoms of *hyperactivity/inattention* (HI), as measured by the HI subscale of the *Strengths and Difficulties Questionnaire* (SDQ) [22] in a large epidemiological cohort. Based on previous findings [17, 18], we hypothesized that symptoms of hyperactivity/inattention are associated with higher voice intensity. Moreover, we explored associations of further voice features including fundamental frequency (f0), jitter, maximum phonation time (MPT) and *Dysphonia Severity Index* (DSI) and HI symptoms based on preliminary findings from earlier research in small samples [17, 18, 23].

## Participants and methods

### Study design and participants

This study analysed data from the *LIFE Child study*. This is an ongoing epidemiological longitudinal cohort study conducted at the *Research Centre for Civilization Diseases* in Leipzig, Germany (for details see [24]). The LIFE Child study protocols adhere to the Declaration of Helsinki. Written informed consent was received from all parents and children over 12 years of age.

Inclusion criteria for our study were children aged between 5 and 18 years, for whom both speaking-voice examination and information on SDQ HI values were available for one or more time points. Data from 1460 children (female: *n* = 716; male: *n* = 744) with a mean age of 10.96 years (range: 5.65–18.22) were included. Participants were either part of the *Health Cohort* (*n* = 1297) or the *Obesity Cohort* (*n* = 163). Examinations took place between the years 2012 and 2015. The number of visits varied between 1 (*n* = 736), 2 (*n* = 462), 3 (*n* = 231), or 4 (*n* = 31).

### Methods

#### Demographic and health data

Demographic and health data were collected either by clinical interviews/questionnaires or clinical examination. All examinations were carried out by trained medical investigators/paediatricians in the child-friendly facilities of the outpatient clinic of the *Research Centre for Civilisation Diseases* in Leipzig. In addition to voice-specific data, child’s age, sex, *Body Mass Index* (BMI), socio-economic status (SES) and pubertal status were included in our study. Pubertal status was included due to missing information on voice change in most participants. The examination methods relevant to this study are described below and in detail in the study protocol [24].

##### Socio-economic status

SES was calculated using a multidimensional index based on information provided by parents on their school education and vocational training, their occupational status, and their net equivalent income. The operationalisation used is based on the *KiGGS* study of the *Robert Koch Institute* [25]. SES scores can range from 3.0 to 21.0, with values indicating low (3.0–8.4), medium (8.5–15.4), or high (15.5–21.0) SES.

##### BMI-standard deviation score (BMI-SDS)

The original BMI value was calculated using the body mass (kilogram) divided by the square of the body height (m^2^) [24]. To ensure comparability between age and sex groups, BMI-SDS (standard-deviation-scores) were calculated using the Kromeyer-Hauschild reference [26].

##### Pubertal status

The assessment of pubertal status as an indicator of physical maturity is based on Tanner’s criteria for pubertal development [27, 28]. For this study, a corrected form of the pubertal status was used. In some cases, Tanner’s criteria did not align with corresponding hormone levels (endocrine values). Certain endocrine values indicated a lower or higher physical maturity. To address this discrepancy, laboratory data were used to adjust the Tanner stage where necessary. Values range from 1 to 5, with higher values indicating greater maturity.

#### Voice measurements and features

Voice measurements were conducted according to a standard operating procedure based on the recommendations by the *Union of the European Phoniatrics* [29] and as described by Berger et al. [30]. Voice examination was carried out in a soundproof room by trained investigators. Voice recording and analysis were conducted using the DiVAS software and a designated self-calibrating microphone headset (XION Medical, Berlin, Germany; Technical specifications of the headset: dynamic range 40–120 dB(A) (sine signal), frequency range 70 Hz–20 kHz, signal-to-noise ratio (SNR) > 62 dB, article number 352 009 010). The software and hardware used are specifically designed for clinical and acoustic voice assessment and are widely applied in clinical voice diagnostics. The head-mounted headset ensured a constant distance of 30 cm between the child’s mouth and the microphone, even when the child turned their head during the examination. This is particularly crucial for accurate measurement of the sound pressure level. Participants had two different tasks. For the analysis of the speaking voice, the task was to count from 21 to 30 at five different sound pressure levels: quietest voice (quiet_I), conversational voice (conversation_II), presentation voice (presentation_III), loudest voice (loud_IV), and quietest voice again (quiet_V) for a voice reset test. In different conditions, children were instructed to speak as soft as possible, but to avoid whispering (I); to speak as if in a normal conversation sitting across from each other (II); to speak as if in a classroom giving a presentation (III), or to shout with their loudest voice, but without screaming (IV). Immediately after the shouting task, the children were instructed to count as quietly as possible again (V). This voice reset test is considered unremarkable if the values are comparable to those of the initial quiet measurement [30, 31]. The second task consisted of sustaining a tone on “na” at a comfortable, medium pitch and volume. After a maximal inhalation, the tone should be sustained for as long as possible. Afterwards, the tone should be sustained once more for a few seconds, aiming for the sound to be as clear and pure as possible.

For the analysis, we included the following parameters: fundamental frequency (f0; Hz) and intensity (dB(A)), extracted from each condition of the first task and maximum phonation time (MPT) and Jitter, extracted from the second task. Additionally, the highest pitch (f0max) and the lowest sound pressure level (SPLmin) achievable with the singing voice were measured. The Dysphonia Severity Index (DSI) was calculated from f0max, SPLmin, MPT, and jitter using a formula by Wuyst et al. [32]. The DSI ranges from + 5 (normal voice) to − 5 (severely dysphonic voice). While normative DSI values are well established for adults [33], there are fewer normative data available for children. Since children’s voices change due to growth and puberty (voice change), DSI values in children are highly variable and age dependent. Compared to adults, children often show slightly lower DSI values. A total of 13 voice-derived features were used for analysis.

#### Behavioural difficulties/strengths

Behavioural data was extracted from the subscale *hyperactivity/inattention* (HI) from the parent-version of the SDQ, a widely used instrument for measuring behaviour in children and adolescents [22]. The subscale HI consists of five questions regarding hyperactivity/impulsivity (e.g. restless/overactive; fidgeting) and inattention (e.g. easily distracted/concentration wanders) on a three-point Likert scale, resulting in scores ranging from zero to ten.

### Statistical analysis

We used two complementary analytical approaches to address different research questions. First, Linear Mixed Models (LMMs) were applied aiming to examine associations between voice features and SDQ HI scores. This approach allowed us to account for the repeated measurements per child and to estimate how specific voice features were related to symptom levels after controlling for relevant covariates. Second, we applied Machine learning (ML) methods to evaluate whether the voice features could be used to predict SDQ HI scores at the individual level. Importantly, the ML analyses predict SDQ HI scores (a proxy of ADHD-related symptoms) rather than a clinical ADHD diagnosis. Results therefore speak to symptom-level associations/predictions and not diagnostic classification. All analyses were conducted using *Python* (3.9), leveraging libraries such as *scikit-learn* [34]. A complete list of libraries and their versions can be found in the public GitHub repository.

#### Linear mixed models

LMM analysis preprocessing included standardising all variables, so they were on the same scale (*z-scaling*). LMMs allowed us to model both fixed effects (voice features, age, sex, SES, BMI-SDS), representing effects that are assumed to be the similar across all children, and random effects, which account for child-specific variation.

A random intercept for each child was included to control for within-subject correlations, and a random slope for the number of visits allowed individual differences in symptom trajectories over time. The main analysis examined whether voice features predicted SDQ HI scores while adjusting for covariates (age, sex, SES, and BMI-SDS). Additional analyses were conducted (a) separately for boys and girls, and (b) including pubertal status as an additional covariate. LMMs were fitted using the python module *statsmodels* and the model’s fit was optimised using a standard procedure (*Powell* method, 1000 iterations). Models were evaluated by calculating the variance of the fixed effects and residuals, reported as coefficients, standard errors, confidence intervals, and p-values. Adjustment for multiple testing was done using the False Discovery Rate (FDR; Benjamini-Hochberg procedure) to reduce the chance of false positives. Results with FDR-corrected p-values (q-values) < 0.05 were considered statistically significant.

#### Machine learning

We conducted a series of ML analyses using *Ridge Regression* (algorithm), that was chosen because it is well-suited for high-dimensional and correlated data, which is typical for voice features. All numerical features were standardized, and where relevant, demographic covariates were regressed out from the voice features (linear confound removal). We trained three models: (1) Full model: voice features + demographics, (2) Demographics-only model, (3) Voice-only model. Comparing models 1 and 2 allowed us to test whether voice features provide added predictive value beyond demographics. Differences in prediction error (Mean absolute error, MAE) were tested statistically using the Nadeau & Bengio corrected t-test, which is specifically developed for cross-validated ML results [35].

The models were evaluated using nested 5-fold cross validation, repeated 20 times. This avoids overly optimistic performance estimates and ensures that the results are stable and not due to random data splitting. The hyperparameters (regularisation strength α, feature set size) were optimally tuned. *GroupKFold* was used to ensure that repeated measurements from the same child did not appear in both training and test sets (thus avoiding data leakage). To evaluate the performance, we calculated the MAE, that reflects average prediction error in SDQ HI points, the amount of explained variation (*R*^2^), and how strongly predictions correlated with actual outcomes (*r).* To determine whether models performed better than chance, permutation tests were applied by repeatedly shuffling the outcome labels and re-training the models. A p-value < 0.05 indicated that observed performance exceeded what could be expected if there were no real association. Finally, to understand which features influenced model predictions, we calculated *Shapley Additive Explanation* (SHAP) which quantify each feature’s contribution to the output.

## Results

### Descriptive statistics

Table 1 shows baseline study, demographic, and health characteristics from all included participants. Descriptive statistics on voice features and the SDQ HI from all visits can be found in Table 2. The exact sample sizes and number of features used for each model are in the LMM and ML Results sections, as these varied with the model specifications.

**Table 1:** Baseline study, demographic and health characteristics

Characteristic	n (%)	Mean/n (SD/%)
Sample	1460 (100)	1460 (100%)
Health cohort		1297 (88.8%)
Obesity cohort		163 (11.2%)
Number of visits	1460 (100)	1.70 (0.81)
1		736 (50.4%)
2		462 (31.6%)
3		231 (15.8%)
4		31 (2.1%)
Age (years)	1460 (100)	10.96 (3.02)
Sex	1460 (100)	
Female		716 (49.0%)
Male		744 (51.0%)
SES	1402 (96.03)	
Low		196 (14.0%)
Middle		856 (61.1%)
High		350 (24.9%)
BMI-SDS	1449 (99.25)	0.24 (1.23)
Health cohort		−0.03 (0.99)
Obesity cohort		2.41 (0.62)
Pubertal status	1205 (82.53)	2.20 (1.52)

*SES* socio-economic status, *BMI-SDS* BMI-standard deviation score

**Table 2:** Characteristics of voice and SDQ HI variables (all visits)

Characteristic	n	Mean (SD)	Range
SDQ			
HI	2418	3.06 (2.47)	0.00–10.00
Voice features			
f0_quiet_I	2418	186.67 (43.63)	80.39–347.03
f0_conversation_II	2418	204.39 (43.62)	89.04–367.50
f0_presentation_III	2417	227.59 (47.56)	87.99–374.36
f0_loud_IV	2411	296.60 (55.73)	124.24–425.38
f0_quiet_V	2370	198.23 (46.78)	90.30–371.00
spl_ quiet_I	2418	51.46 (3.17)	41.62–65.14
spl_conversation_II	2418	58.93 (3.29)	48.56–73.56
spl_presentation_III	2417	65.88 (4.16)	54.41–86.86
spl_ loud_IV	2411	81.47 (6.30)	48.45–102.82
spl_ quiet_V	2370	54.06 (3.53)	43.85–82.51
Jitter	2357	1.89 (3.49)	0.13–42.65
MPT	2357	13.05 (5.01)	1.59–36.32
DSI	2357	1.77 (4.42)	−46.74–10.03

*SDQ* Strengths and Difficulties Questionnaire, *HI* subscale hyperactivity/inattention, *f0* fundamental frequency (Hz), *spl* sound pressure level (intensity; dB(A)), *MPT* maximum phonation time, *DSI* dysphonia-severity-index

### Correlation analysis: relevant covariates

**Fig. 1:**
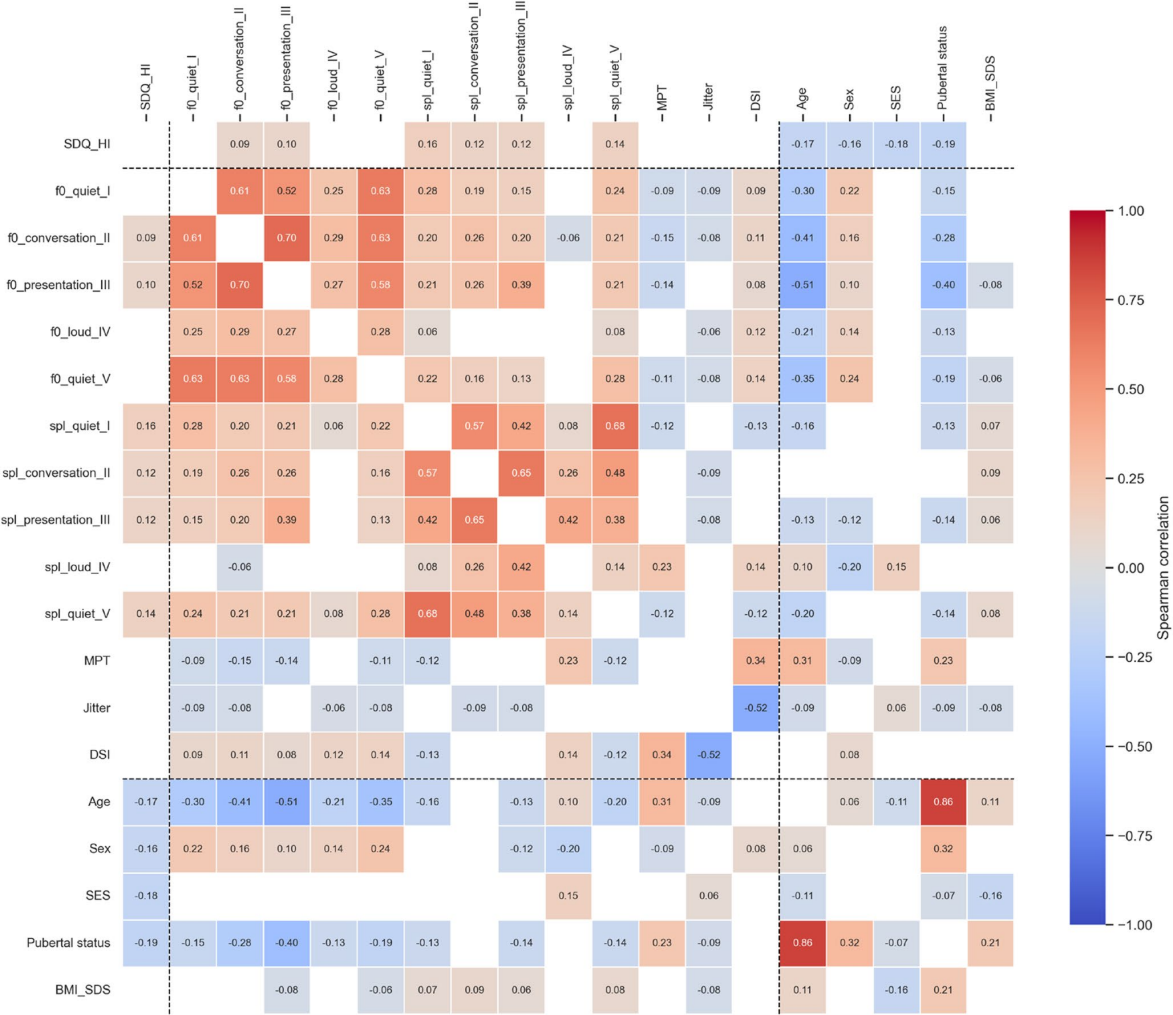
Correlation matrix of significant correlations after FDR-correction (Spearman’s Rho). Note. Only statistically significant correlations (FDR-corrected p < 0.05) are displayed in the figure; SDQ HI = Strengths and Difficulties Questionnaire subscale hyperactivity/inattention; f0 = fundamental frequency; spl = sound pressure level

Covariates included age, sex, SES, BMI-SDS and pubertal status. They were selected based on previously reported associations with the SDQ [36, 37] or voice features [38], and the results of a Spearman-Rho correlation analysis (see Fig. 1). To handle multicollinearity, highly correlated predictors (*r* > 0.8) were not analysed in the same LMM model; therefore, age and pubertal status were analysed in separate models.

### Linear mixed models

#### LMM: main analysis

**Fig. 2:**
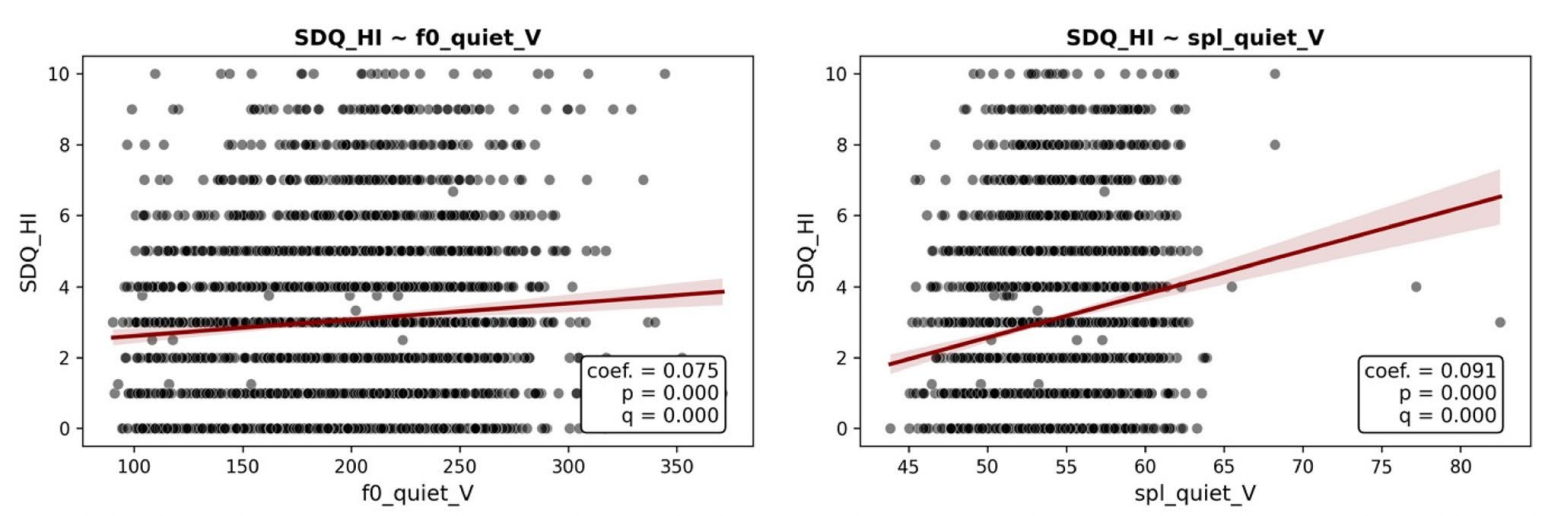
Scatterplot SDQ HI and f0/spl_quiet_V. Note. SDQ HI = Strengths and Difficulties Questionnaire subscale hyperactivity/inattention; f0 = fundamental frequency; spl = sound pressure level (intensity); coef = coefficient; p/q = p-/q-value (FDR adjusted *p*-value)

The main LMM analysis revealed that several voice variables were significantly associated with SDQ HI values (Table 3). The total number of included values per voice feature (*n*) varied between 2370 and 2418, due to data availability for the different conditions. Including age, sex, SES and BMI-SDS as covariates, and correcting for multiple testing, fundamental frequency (f0) in conditions 2–5 and voice intensity (spl) in all conditions were found to be significantly associated with HI values. These associations indicated that higher SDQ HI values corresponded with a voice that was used louder and at a higher pitch during the counting task. The largest effects were detected in the *voice reset test* (quiet_V, speaking quiet again) for both f0 (*β* = 0.075, *q* < 0.001) and intensity (*β* = 0.091, *q* < 0.001) (Fig. 2). No significant effects were found for other voice features, namely Jitter (*β* = 0.024, *q* = 0.104), MPT (*β* = −0.019, *q* = 0.332), and DSI (*β* = −0.022, *q* = 0.152).

**Table 3:** Significant associations between SDQ HI and voice variables (linear mixed model)

SDQ	Voice feature	*n*	df_resid	coef (*β)*	se	ci_lower	ci_upper	p	q
HI	f0_conversation_II	2418	2412	0.070	0.019	0.033	0.106	< 0.001	< 0.001
	f0_presentation_III	2417	2411	0.057	0.020	0.018	0.096	0.005	0.009
	f0_loud_IV	2411	2405	0.037	0.016	0.006	0.068	0.021	0.029
	f0_quiet_V	2370	2364	0.075	0.017	0.041	0.109	< 0.001	< 0.001
	spl_ quiet_I	2418	2412	0.070	0.016	0.038	0.101	< 0.001	< 0.001
	spl_ conversation_II	2418	2412	0.054	0.016	0.023	0.086	0.001	0.002
	spl_ presentation_III	2417	2411	0.037	0.016	0.006	0.068	0.018	0.026
	spl_ loud_IV	2411	2405	0.049	0.016	0.017	0.081	0.002	0.005
	spl_ quiet_V	2370	2364	0.091	0.016	0.059	0.123	< 0.001	< 0.001

All models were adjusted for the covariates age, sex, SES, BMI-SDS; all models converged successfully; degrees of freedom for all models are 9; SDQ HI = Strengths and Difficulties Questionnaire subscale hyperactivity/inattention; f0 = fundamental frequency; spl = sound pressure level (intensity); df_resid = degrees of freedom (residual); coef = coefficient; se = standard error; ci_lower/upper = confidence interval (lower/upper bound); p/q = p-/q-value (FDR adjusted *p*-value)

To confirm that the findings were stable, we repeated the analysis with alternative model setups. The robustness analysis, which included puberty status instead of age, indicated stable main effects with an overall consistent pattern of the results (see Online Resource 1.1). The results of the sex-stratified analysis also demonstrated a similar pattern (see Online Resource 1.2).

### Machine learning

The main analysis was performed using 2314 values per feature obtained from 1433 children. Averaged over all repetitions and folds, the model including voice features and covariates (main model) showed an *MAE* of 1.869, an *R*^2^ of 0.120 and an *r* of 0.356, revealing a moderate relationship between predicted and true SDQ HI scores. Permutation testing (1000 iterations) confirmed significance (*MAE (true)* = 1.864, *MAE* (*random*) = 2.016 *p* < 0.001). Comparing the *MAE*’s from the main model to a model using only covariates (age, sex, SES, BMI-SDS) revealed superior performance of the model including voice features (*t* = 1.868; *p* = 0.032). Permutation testing (1000 iterations) for the model that used only voice features was significant (*MAE* (true) = 1.982, *MAE* (random) = 2.015, *p* < 0.001), confirming that the model performed better than chance.

**Fig. 3:**
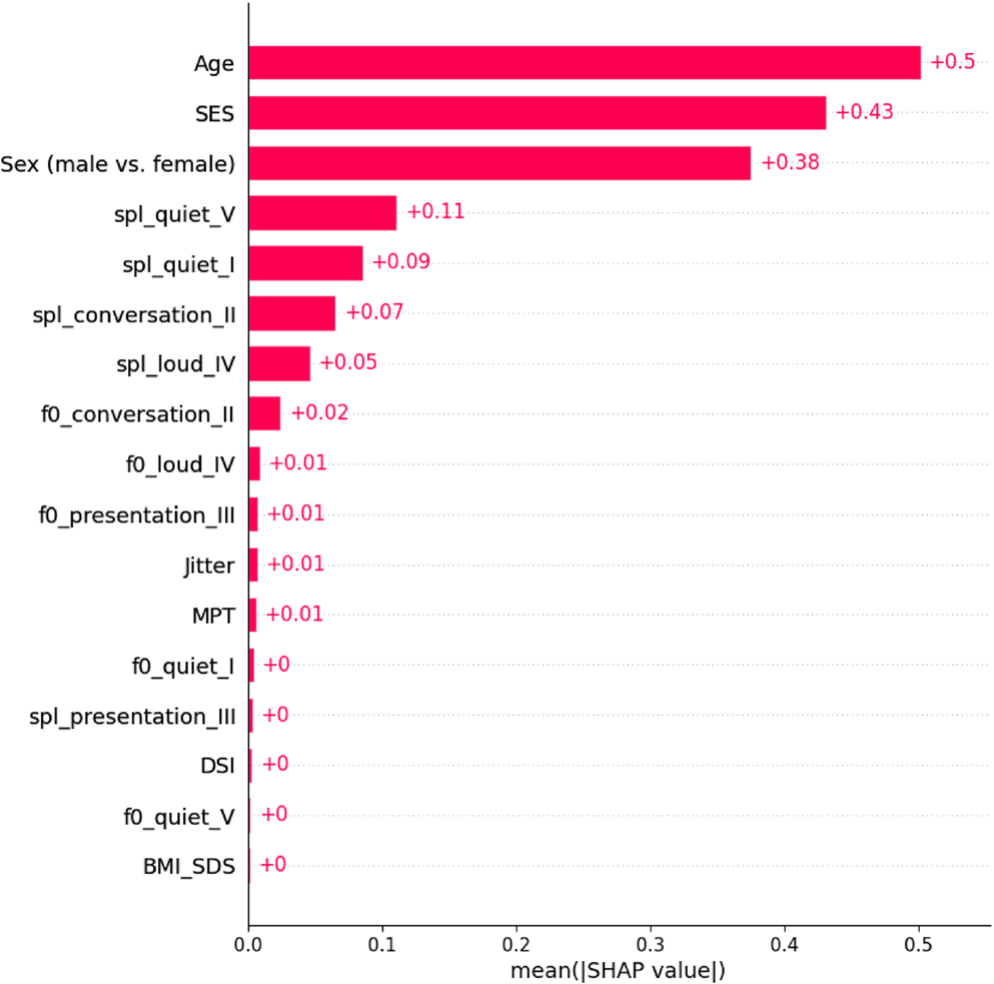
SHAP bar plot main analysis (Ridge Regression). Note. SHAP = Shapley Additive Explanation; SES = socio-economic status

Feature importance was assessed using SHAP, which estimates how much each feature contributes to the model’s predictions. For the main analysis, age, SES and sex are the main predictors for SDQ HI (Fig. 3). Several voice features contributed meaningfully to the predictions. Especially the intensity values of the *voice reset test*, i.e. *spl_quiet_V*, and the *quiet_I* condition seemed to be important voice features, aligning with the results of the LMM.

**Fig. 4:**
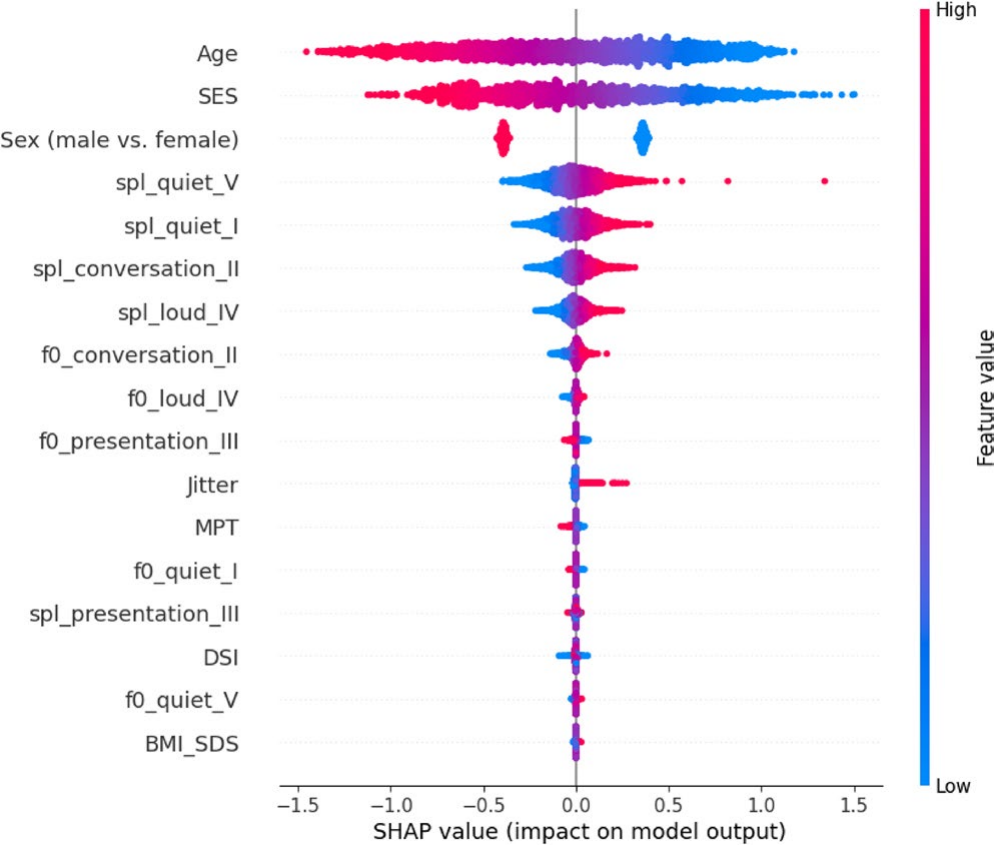
SHAP dot plot main analysis (Ridge Regression). Note. SHAP = Shapley Additive Explanation; SES = socioeconomic status; MPT = Maximum phonation time; DSI = Dysphonia-severity-index; BMI_SDS = BMI-standard deviation score

A SHAP dot plot was used to assess the influence of each feature on the model’s prediction, each dot representing one participant and the colour indicating whether the feature’s value was high or low. The results of the SHAP dot plot (Fig. 4), showed that age and SES have the broadest spread, suggesting relatively strong influences on the predicted outcome. Having a lower age or SES, and being male, increased the predicted SDQ HI scores. Speaking at a higher intensity during the quiet conditions also increased the predicted SDQ HI scores. Jitter had a small average effect on prediction but showed a relatively wide horizontal spread. A wide horizontal spread indicates substantial variability in how strongly that feature influences predictions across individuals.

#### ML: impact of pubertal status

A robustness analysis was performed using 1903 values per feature obtained from 1248 children. Results confirmed that the models’ predictions were reliable even when pubertal status was included as additional covariate (*MAE* = 1.902; *R*^2^ = 0.124; *r* = 0.361). The result of the permutation test was significant (*p* < 0.001). SHAP values verified that demographic variables (i.e. SES, sex, pubertal status, age) were still the most important predictors of SDQ HI. The analysis reaffirmed the voice intensity during the quiet conditions, i.e. *spl_quiet_I/_V*, as a moderately important predictor.

#### ML: sex-stratified analysis

Averaged *MAE* values from the main analysis were calculated and separated by sex (female: *MAE* = 1.749; male: *MAE* = 1.974). A *Mann–Whitney-U-Test* revealed significant differences between boys and girls (*U* = 731192.0; *p* < 0.001) indicating better performance in girls. However, after z-standardizing the SDQ HI scores within each sex, model performance was virtually identical for both groups (female: *MAE* = 0.751; male: *MAE* = 0.778) indicating that the observed differences are attributable to distributional differences in SDQ HI, not true sex-specific model effects.

Using two separate models the sex-stratified analysis of girls was performed using 1109 values per feature obtained from 707 girls and 1205 values per feature from 726 boys. Sex-stratified analyses showed again lower averaged *MAE* for girls (*MAE* = 1.746, *R*^2^ = 0.101, *r* = 0.337) compared to boys (*MAE* = 1.985, *R*^2^ = 0.083, *r* = 0.306), with both models reaching statistical significance in permutation testing (*p* < 0.001).

**Fig. 5:**
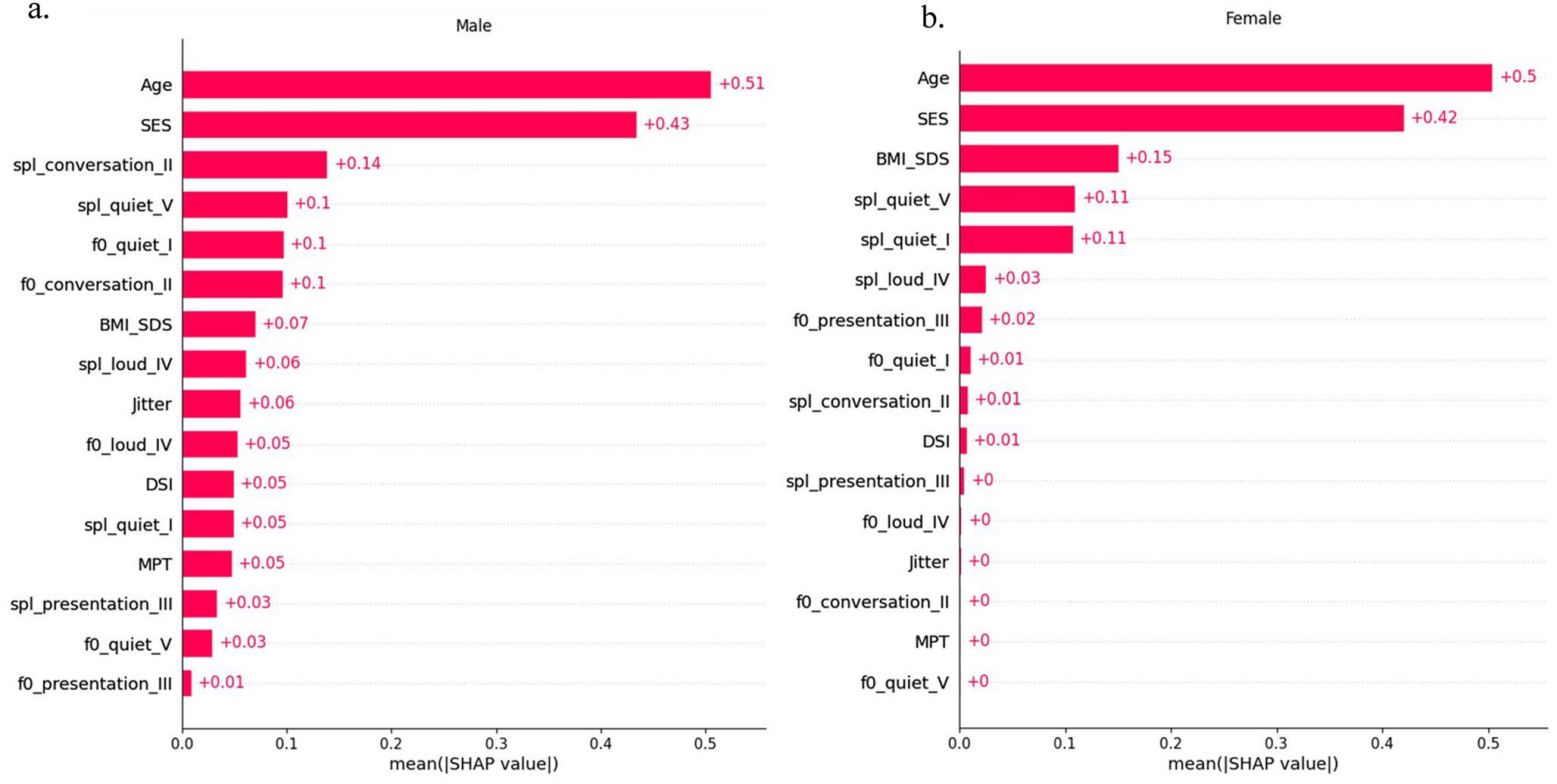
SHAP bar plot sex-stratified analysis (Ridge Regression). Note. a = Boys, b = Girls; SHAP = Shapley Additive Explanation; SES = socio-economic status

Averaged absolute SHAP values indicated apparent sex-specific differences in predictive patterns. Although demographic variables remain to be the main predictors, namely age, SES and BMI-SDS in females, different voice features contribute to the prediction of SDQ HI in boys and girls (Fig. 5). The model for boys indicated that besides voice intensity (condition II, V) f0 in the quiet I condition appeared important in predicting male SDQ HI. For the girls, voice intensity in the quiet conditions (I, V) seemed to be the most important voice feature.

To further examine potential sex-specific predictive effects of voice features, we included sex-by-voice feature interaction terms in the main model. This means that specific model terms were included to enable testing of how the effect of each voice feature changed depending on sex. Although several sex-by-voice feature interactions were selected during model fitting, residual analyses showed no statistically significant differences in model performance between boys and girls. These results indicate that, despite apparent sex differences in feature importance, the predictive value of voice features for SDQ HI is not sex-specific when controlling for general differences in voice characteristics between boys and girls. Details are provided in Online Resource 2.

## Discussion

To our knowledge, this is the first study to analyse the potential of voice markers in children and adolescents for diagnostic purposes using machine learning in a large epidemiological sample. In LMM, we identified voice pitch (f0) and intensity, to be positively associated with SDQ HI values over different voice tasks. Using a ML approach, we found that voice features, along with age, sex, and SES, moderately predicted SDQ HI scores. Our sex-stratified analyses showed that robust prediction can be achieved with mixed-sex samples, as model performance was virtually identical for boys and girls after adjusting for variance. These findings support the use of mixed-sex samples enabling larger and more representative data collection. Together, our results provide a sound basis for further investigation of voice features as potential objective markers in the diagnostic process of ADHD in youth.

LMMs showed that children with higher SDQ HI scores exhibited higher voice intensity across all conditions and higher f0 values in conditions 2–5. This positive association indicated that children who showed more hyperactive/inattentive behaviour also display altered voice characteristics in the form of a louder/higher voice. Of note, the strongest effects were found in the *voice reset test*. This suggests that children with more pronounced symptoms of hyperactivity/inattention might find it more difficult to speak quietly once they have spoken normally/loudly. Our findings are consistent with previous studies suggesting that children with symptoms of ADHD tend to show hyperfunctional vocal behaviour, e.g. speaking louder than controls [17, 18, 23]. In contrast to the results of Hamdan et al. [23] and Garcia-Real et al. [18], we found that f0 tended to be higher, not lower, in children with higher SDQ HI scores. The positive correlation between f0 and intensity is consistent with their physiological linkage. Both are influenced by vocal effort or subglottal pressure, reflecting i.e. how strongly air is pushed from the lungs through the vocal folds during voice production [39, 40]. Chronic vocal misuse, for example permanently speaking too loud or with increased pitch could lead to dysphonia [41]. Within this phenomenon, vocal fold nodules (VFN) are the most common diagnosis [42]. Previous research showed that hyperactive behaviour might be an important risk factor for the development of VFN [43]. These are the consequences of sustained tension and high air pressure beneath the vocal folds, which can put strain on the delicate tissue that covers them. However, voice characteristics should be understood as behavioural and physiological correlates of ADHD-related traits, rather than as direct indicators of the disorder itself. While dopaminergic alterations associated with ADHD may influence motor processes involved in voice production, these effects likely represent downstream physiological sequelae rather than primary signatures of ADHD pathophysiology. Thus, voice features may serve as indirect markers of ADHD-related behaviour, but not as direct neurobiological indicators of the disorder.

No significant associations were found between Jitter, MPT, or DSI and SDQ HI. Based on previous findings, Jitter values seem to be higher in children with ADHD or dysphonia [18, 44]. In addition, MPT appears to be shorter in children with dysphonia in general or with VFN specifically [45]. There is currently no study linking hyperfunctional/inattentive behaviour to the DSI, although this index is used to assist in the diagnosis of dysphonia [46]. So far, only a few comprehensive normative values for the Dysphonia Severity Index (DSI) exist for children and adolescents. Children tend to have lower DSI values than adults - for example, 7- to 9-year-olds in Shanghai showed lower values than 18- to 23-year-olds [47]. Among Indian children, girls had significantly higher DSI values (mean 3.8 vs. 2.9), whereas no sex differences were found in the Shanghai cohort [48]. The DSI values we measured fall below this range, thus indicating a level that may suggest mild pathological changes in the voice.

Our ML model moderately predicted SDQ HI scores using voice features and relevant demographic information, such as age and sex, as covariates. Voice features alone yielded significant predictive power and further enhanced model performance when combined with covariates, suggesting a biologically plausible, independent contribution to the prediction of hyperactivity/inattention symptoms. This aligns with previous studies describing the potential of voice features as diagnostic markers, including psychiatric conditions [11, 49].

SHAP analyses highlighted that voice intensity in quiet conditions contributed meaningfully to the prediction, again suggesting difficulty in modulating volume with more prominent symptoms [17, 18, 23]. While the Jitter value appears to have minimal influence on the prediction, an examination of the SHAP dot plot revealed a relatively large horizontal distribution of this feature. This indicated that high Jitter values resulted in increased predicted HI values, which is in line with findings of previous studies [18, 44]. Jitter is an acoustic index for variations in the fundamental frequency, measuring micro-variations in the periodic sound signal [46]. Among other parameters, Jitter can be used to detect the presence of a voice disorder, where higher values indicate greater variability or more irregularity in vocal fold vibration [46]. A study with 237 Egyptian children aged 4 to 12 years aimed to establish a prototype database of normal acoustic parameters. The mean jitter percentage was reported as 1.9% in boys and 1.6% in girls, with no significant sex differences observed. The study also noted a decline in jitter percentage with increasing age [50]. A prior study suggested alterations of Jitter in children with ADHD, possibly due to lower control stability of vocal folds, resulting in a less periodic larynx vibration [18].

Although initial sex-stratified analyses suggested better performance of the ML model in girls, this was attributable to lower variance in SDQ HI scores rather than true model differences. After adjusting for variance, predictive accuracy was nearly identical across sexes. This is clinically encouraging, as girls—despite being underdiagnosed—were not disadvantaged by voice-based models. While SHAP analyses in both sex-stratified and interaction models revealed sex-specific feature contributions, residual analyses showed no sex differences in model performance. This suggests that observed SHAP differences reflect normative sex-based variation in voice characteristics, rather than distinct predictive mechanisms requiring sex-specific models. Sex-specific models may therefore be unnecessary, though sex remains an important lens for interpretation and validation. In summary, sex-stratified analyses suggests that moderate prediction can be achieved using mixed-sex samples without disadvantaging either sex. In turn, this supports the use of large, diverse samples to enhance generalizability and statistical power in future studies.

In line with earlier research [36, 38], our findings confirmed that key demographic variables such as age, sex, and SES are associated with both SDQ HI scores and voice features. Including these covariates in our models significantly improved predictive performance, underscoring the importance of accounting for known confounders when using voice-based prediction. This supports van Dellen’s [4] claim that predictions should not rely solely on outcome markers, but must consider static, dynamic and contextual factors (e.g. familial, social and cultural), that shape the clinical picture. Similarly, Tan and Benos [51] emphasise the need for ML diagnostics to incorporate information that shapes a patient’s health outcome, including demographic factors, to build accurate models that reflect the complexities of the real world.

### Strengths and limitations

Strengths of the study include a highly standardised voice acquisition, thereby ensuring good data quality. The large sample size, alongside rigorous validation procedures allowed us to apply machine learning with substantially reduced risk of overfitting. Importantly, both analytical approaches converged: the LMMs identified intensity and f0 as associated with SDQ HI, and the ML models independently highlighted the same features through SHAP. This convergence across methods increases confidence in the robustness of the findings.

Limitations pertain to the limited set of voice features that were included in this analysis. While serial speech (reciting number words) is a well-standardized task for children and adolescents, it does not reflect the characteristics of everyday communication. Therefore, additional investigations using standardized texts (e.g., the Rainbow Passage) and recordings of spontaneous speech are warranted in future studies. Some voice features, such as jitter and shimmer, are sensitive to recording conditions and may therefore be affected by them. Although quality control steps were applied, like a soundproof room and a standardized mouth to microphone distance, the results in regard to jitter and shimmer should be interpreted with caution and in light of this potential variability.

Additionally, a clear limitation of the study is the use of the SDQ HI to measure ADHD. The outcome of this scale acts more as a proxy for ADHD and may not capture the full clinical picture. Furthermore, concerns have been raised that the predictive algorithm of the SDQ is not sensitive enough to screen for ADHD [52], as well as calling into question the feasibility of screening the general child population for ADHD using only the parent SDQ HI subscale [53]. Lastly, the outcome of the SDQ HI is a composite score that captures all dimensions of ADHD, making it harder to distinguish which symptoms, such as inattention or hyperactivity, have a greater or lesser impact on voice characteristics. Beyond content validity, measurement reliability constrains achievable prediction: in general, the maximum expected correlation with any external predictor is bounded by the square root of the target’s reliability (attenuation). Thus, any underperformance partly reflects noise in SDQ HI rather than a lack of signal in the voice features. Moreover, SDQ HI aggregates two inattention items, two hyperactivity items and one impulsivity item; if voice features relate differentially to these facets, aggregation may dilute effects. Future work should analyse ADHD factors (e.g., inattention, hyperactivity/impulsivity) separately and include multi-informant reports (parents/teachers) and clinical diagnoses to improve construct alignment.

## Conclusion

Within the framework of precision psychiatry, the results of this study provide first insights into the identification and potential use of voice markers in children and adolescents. Our results indicate the added value of voice features to the mostly subjective evaluation of symptoms of hyperactivity/inattention and stress the need of further investigating the potential of voice-based ML models to detect childhood psychiatric disorders. Research on adults has already confirmed voice features as potential diagnostic markers in psychiatric populations [54]. In adult ADHD, previous research has shown that vocal features, including f0 and jitter, can be used to classify subtypes [55]. The prospect that paediatric voice features could also be used as markers to support a more accurate diagnostic process remains to be explored but seems promising. In summary, we identified voice markers with predictive value in the general population, warranting further investigation in clinical samples.

## Supplementary Information

Below is the link to the electronic supplementary material.

Supplementary Material 1 (XLSX 19.1 KB)

Supplementary Material 2 (PDF 441 KB)

## Data Availability

The data that support the findings of this study are not openly available due to reasons of sensitivity. The codes used for statistical analyses are available in the following GitHub repository: https://github.com/RaBa-fzj/VoicesOfChange.git.
