# Supplementary material for: Voices of change: associations between vocal markers and symptoms of ADHD - Findings from the LIFE child study
Source: Eur Child Adolesc Psychiatry. 2026 Feb 26;35(6):1855–66. doi: 10.1007/s00787-025-02954-9 (PMC13337620; doi:10.1007/s00787-025-02954-9)
Supplement: Supplementary file 2 — Supplementary Material 2 (PDF 441 KB) [file 787_2025_2954_MOESM2_ESM.pdf]

Online Resource 2

Voices of change: Associations between vocal markers and symptoms of ADHD - Findings from the LIFE Child Study. European Child & Adolescent Psychiatry. Bamberger, R., Lotter, L.D., Nieto, N., Poulain, T., Körner, A., Kiess, W., Fuchs, M., & Von Polier, G. Corresponding author: Rachel Bamberger (Research Centre Jülich, INM 7), r.bamberger@fz-juelich.de

2 Sex-stratified interaction analyses

To further assess the role of sex-by-voice feature interactions in predicting SDQ HI, we extended the main model to include all possible sex*voice interaction terms and applied model selection (*SelectKBest*) within a nested cross-validation framework. While several sex-by-voice interaction terms were selected during model fitting (see table S2.1) - reflecting underlying biological differences in voice features between sexes - overall model performance (MAE) did not improve compared to the model without interactions. This suggests that, while these interaction effects may capture meaningful sex differences in voice characteristics, they do not add predictive value beyond the main effects of voice features and sex.

Table S2.1. Top features and interaction terms (sex × voice feature) with mean absolute SHAP value >0.1

Feature	Mean Absolute SHAP Value
Age	0.42
SES	0.41
f0_condition_1_x_sex	0.39
f0_condition_3_x_sex	0.30
f0_condition_4_x_sex	0.26
spl_condition_2_x_sex	0.16
f0_condition_1	0.14
spl_condition_1_x_sex	0.13
spl_condition_5_x_sex	0.12
f0_condition_2_x_sex	0.12
f0_condition_2	0.11
f0_condition_5_x_sex	0.11
spl_condition_4_x_sex	0.10

Note. f0 = fundamental frequency; spl = sound pressure level (intensity).

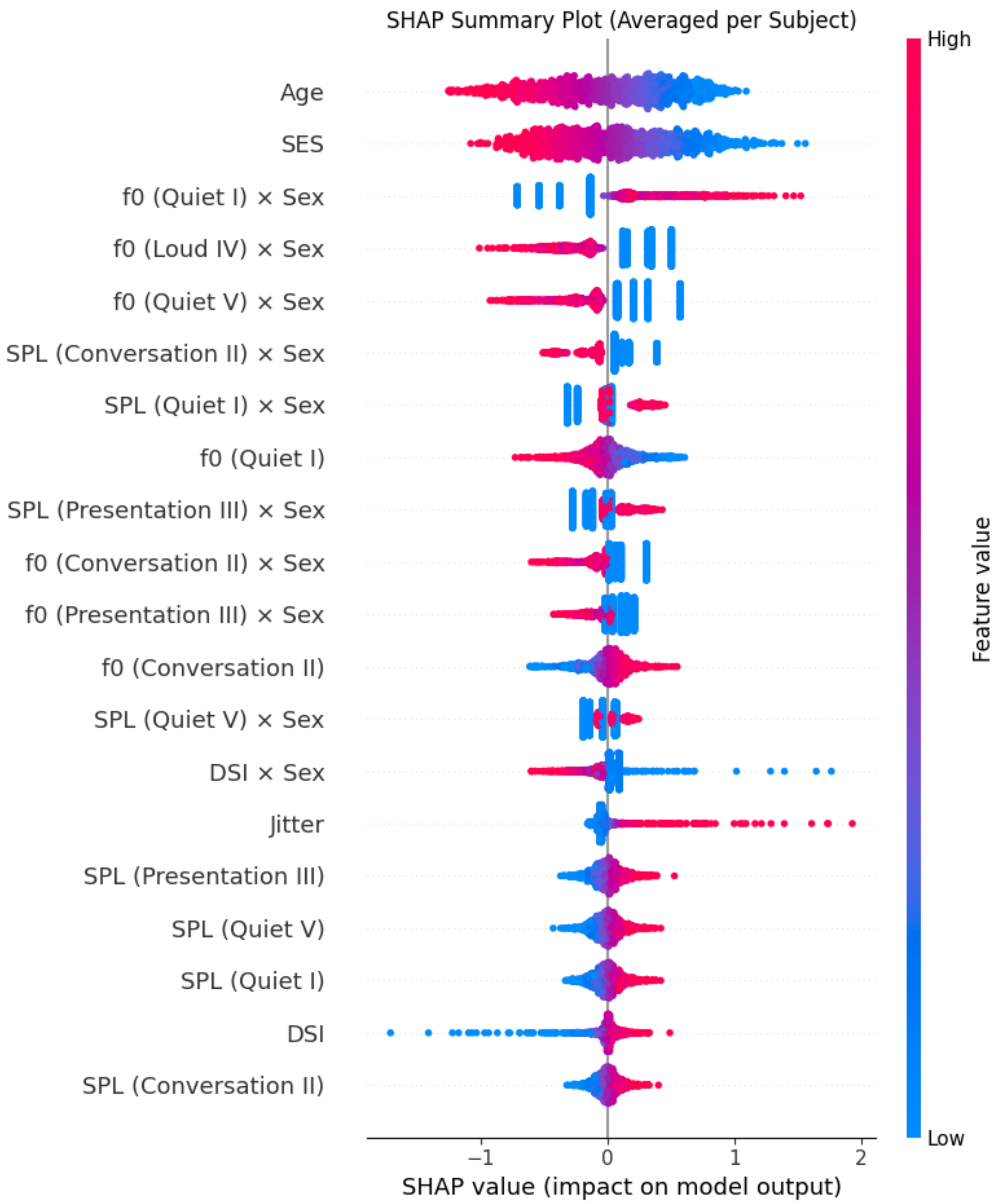

Figure S2.1. SHAP summary plot for the full model including sex-by-voice feature interactions. Each point represents the mean SHAP value for a given feature and participant, summarizing the relative importance and direction of each feature's contribution to model predictions across the entire sample. Color indicates the feature value (blue: low, pink: high). Features are ordered by overall importance. f0 = fundamental frequency; spl = sound pressure level (intensity); DSI = dysphonia-severity-index; SES = socio-economic status.

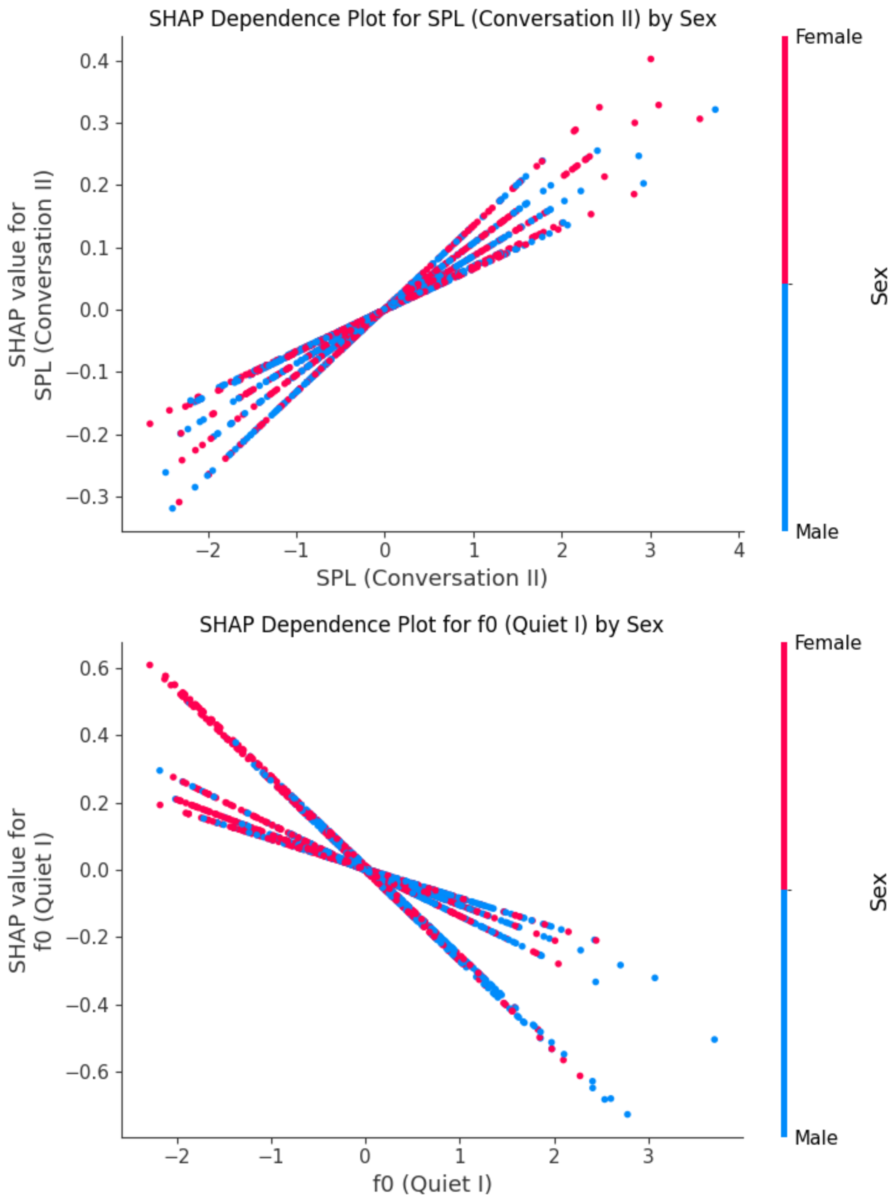

Figure S2.2. SHAP dependence plots for two representative voice features stratified by sex; top: SPL (intensity) in conversational speech; bottom: f0 (fundamental frequency) in quiet speech. Although male and female participants show expected distributional differences in feature values (X-axis), the SHAP values (Y-axis) are largely overlapping, indicating similar contributions to model predictions across sexes.

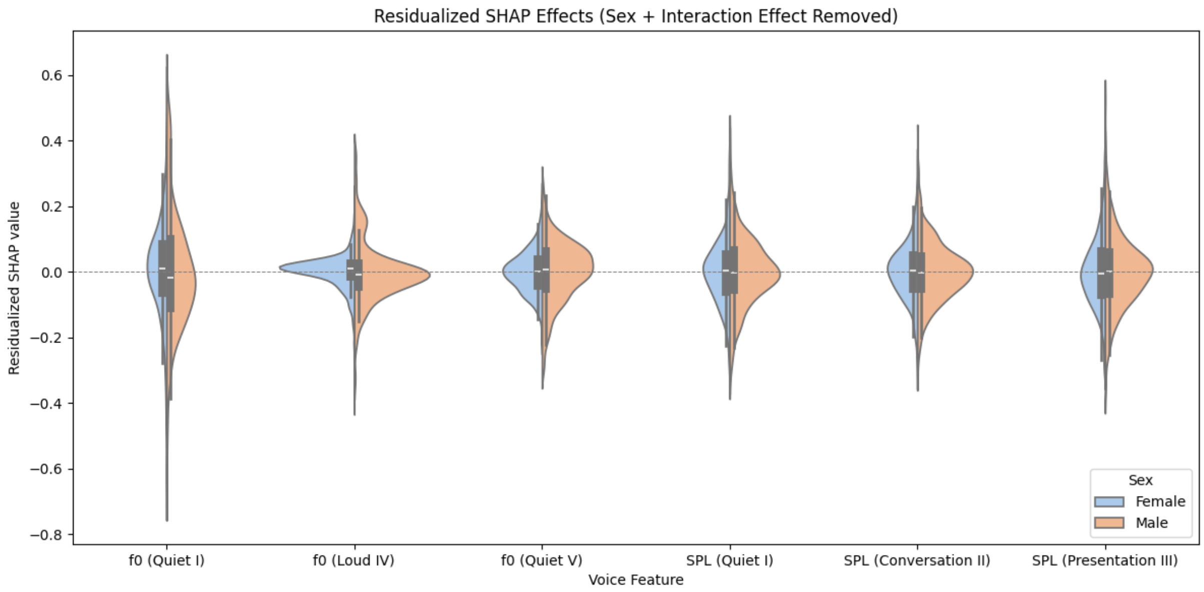

Figure S2.3. Residualized SHAP values of voice features after regressing out sex and its interaction with the respective feature. The figure displays the distribution of residualized SHAP values for voice features with relevance in interaction analyses (see shap summary plot, Fig. S2.2), separated by sex. For each voice feature, the original SHAP values were regressed onto the main effect of sex and an interaction term (voice feature value × sex). The residuals of these regressions represent the portion of the SHAP values not explained by these linear sex-related effects to explore effects beyond biological differences in voice features among sex. The results indicate higher variance in boys, likely due to the higher variance in SDQ HI values in boys but equally distributed mean residualized shap-values. f0 = fundamental frequency; spl = sound pressure level (intensity).
